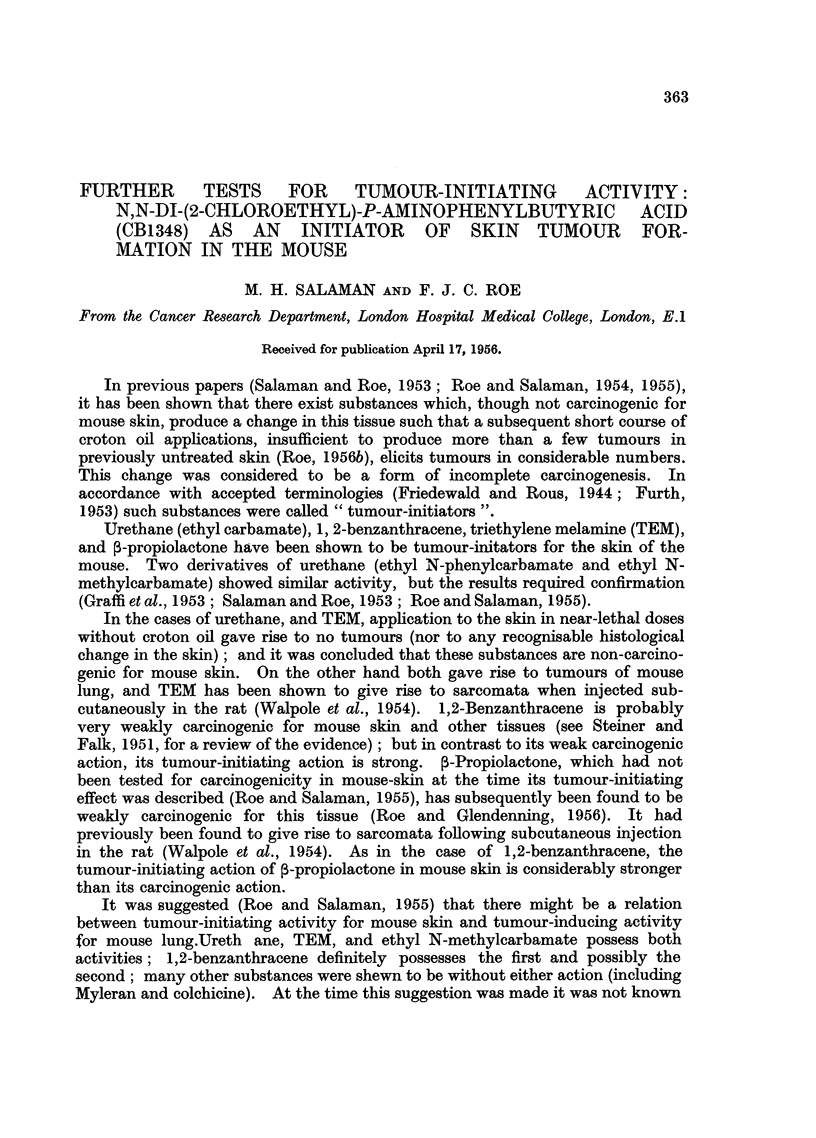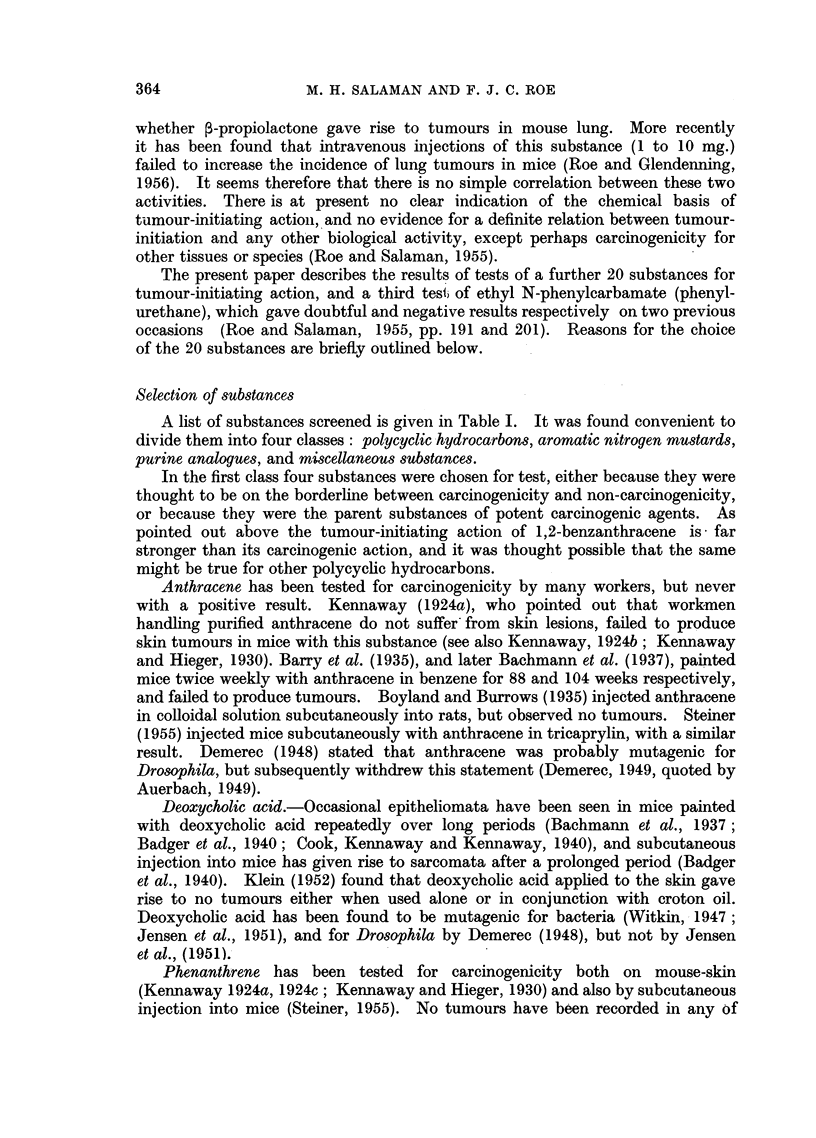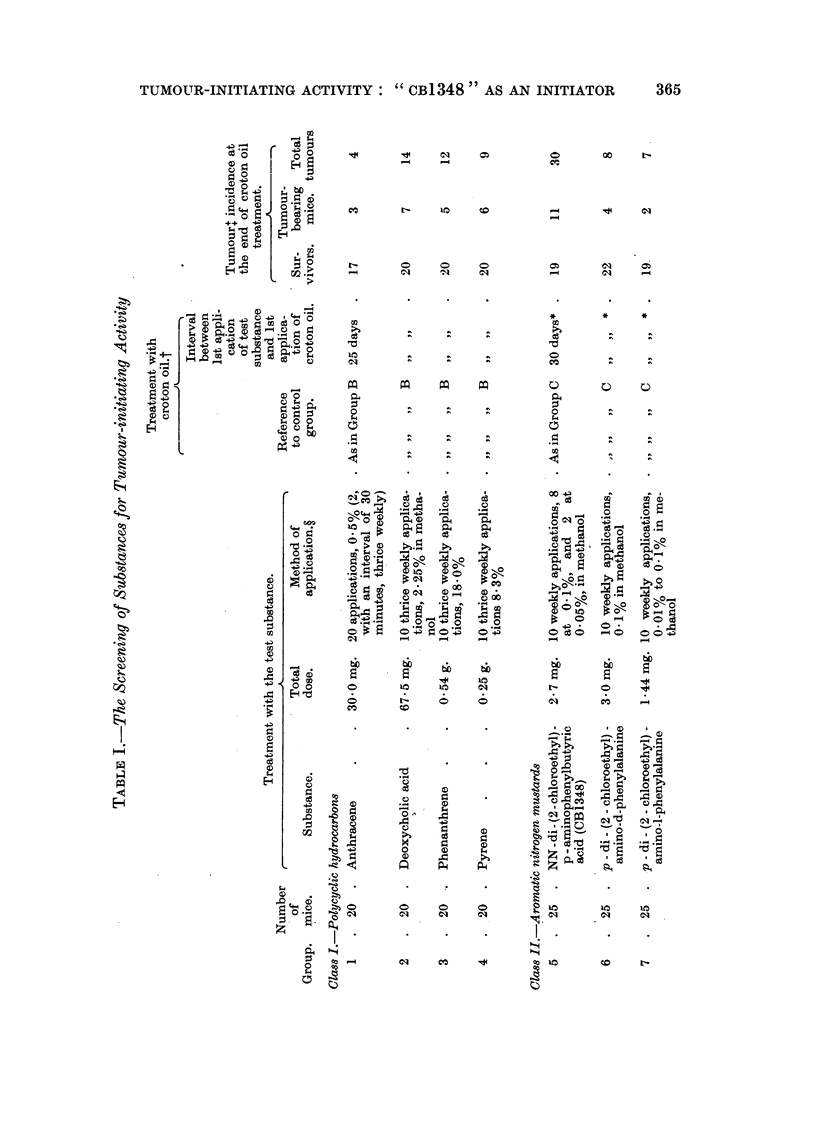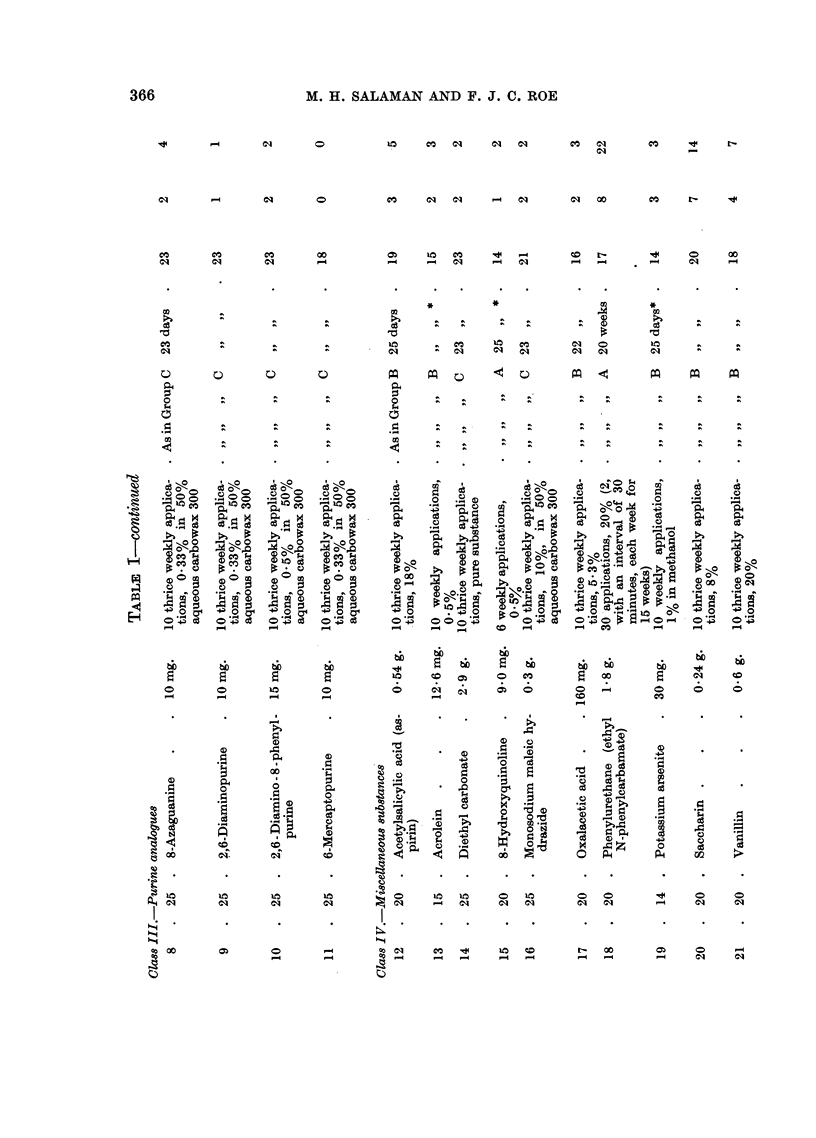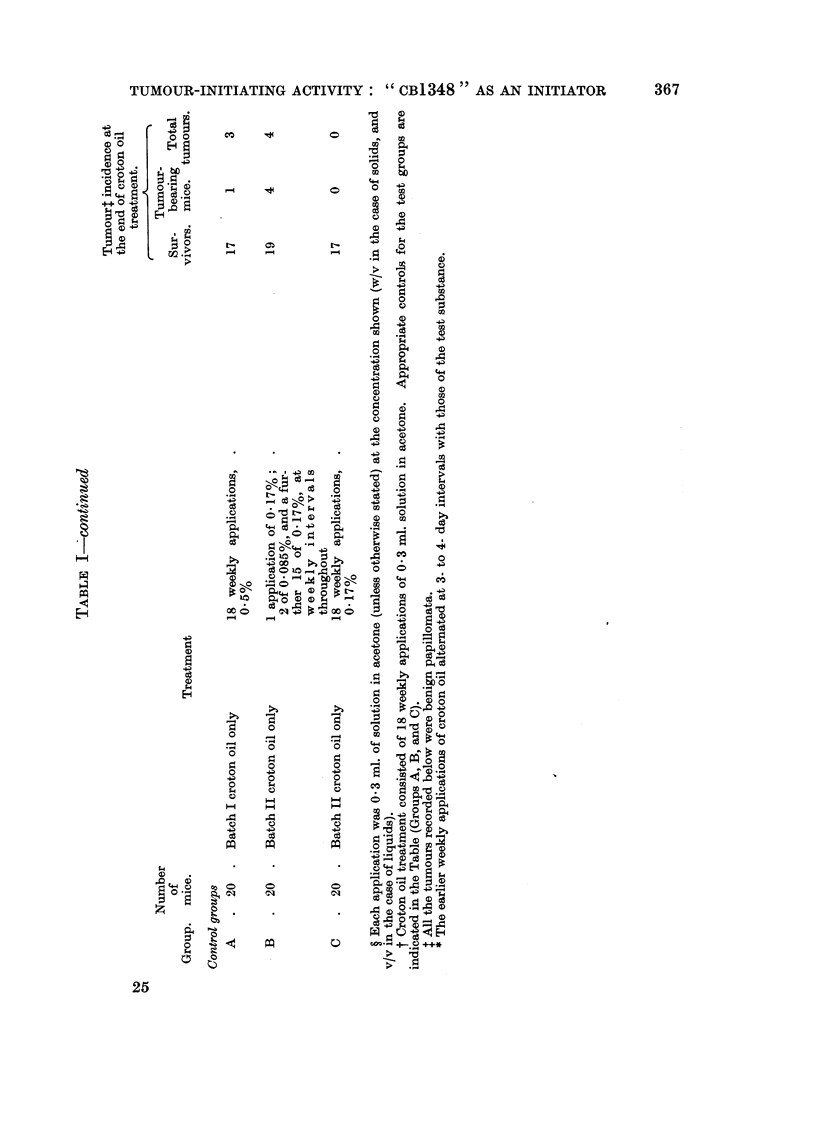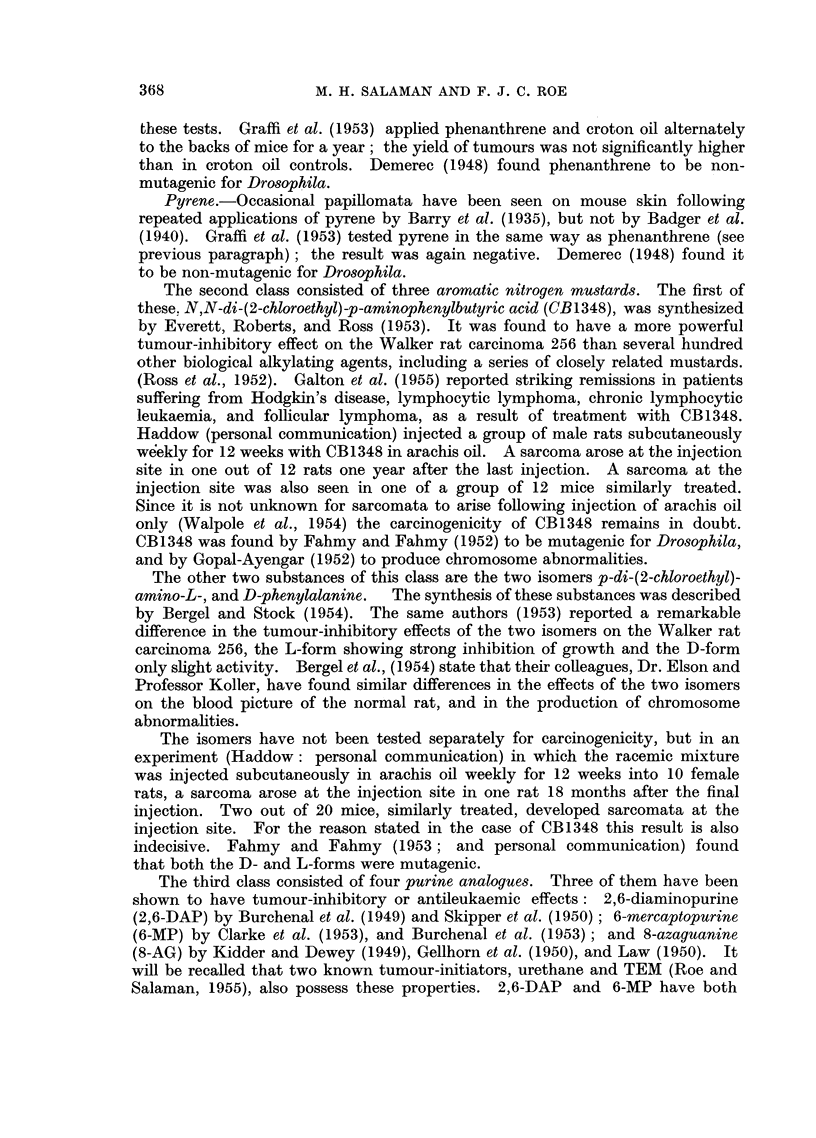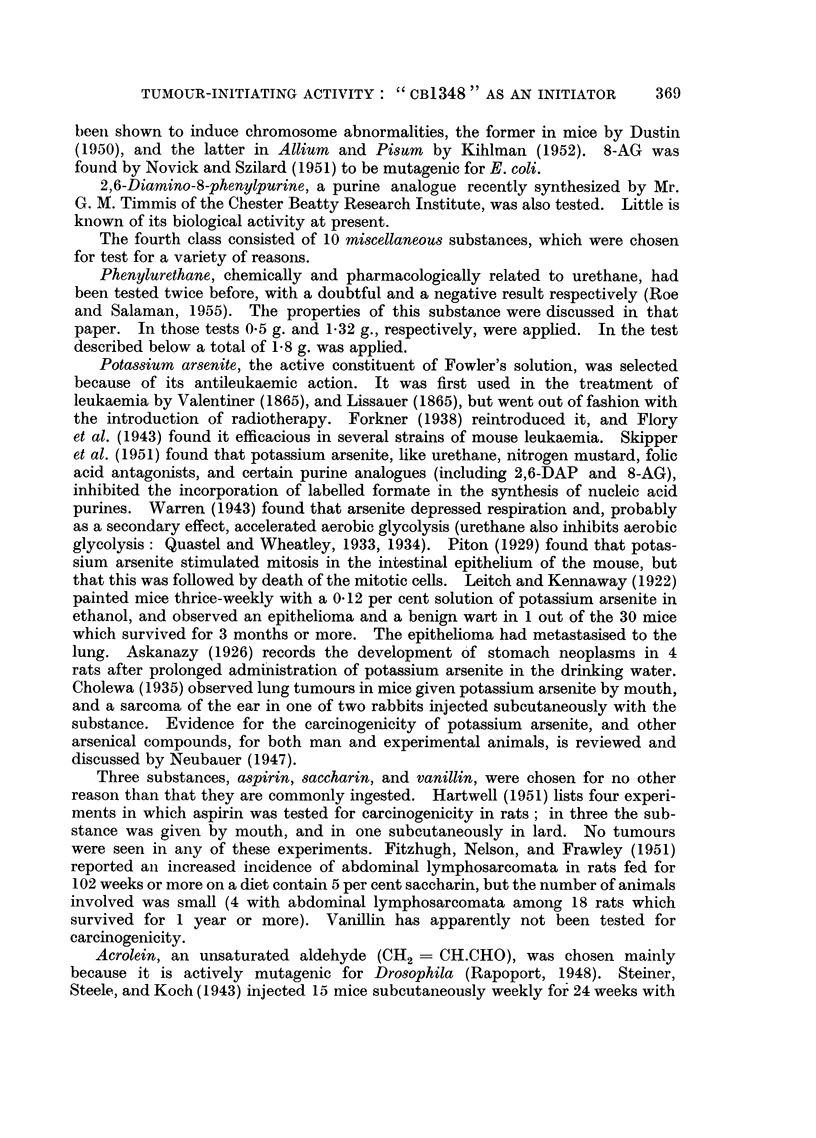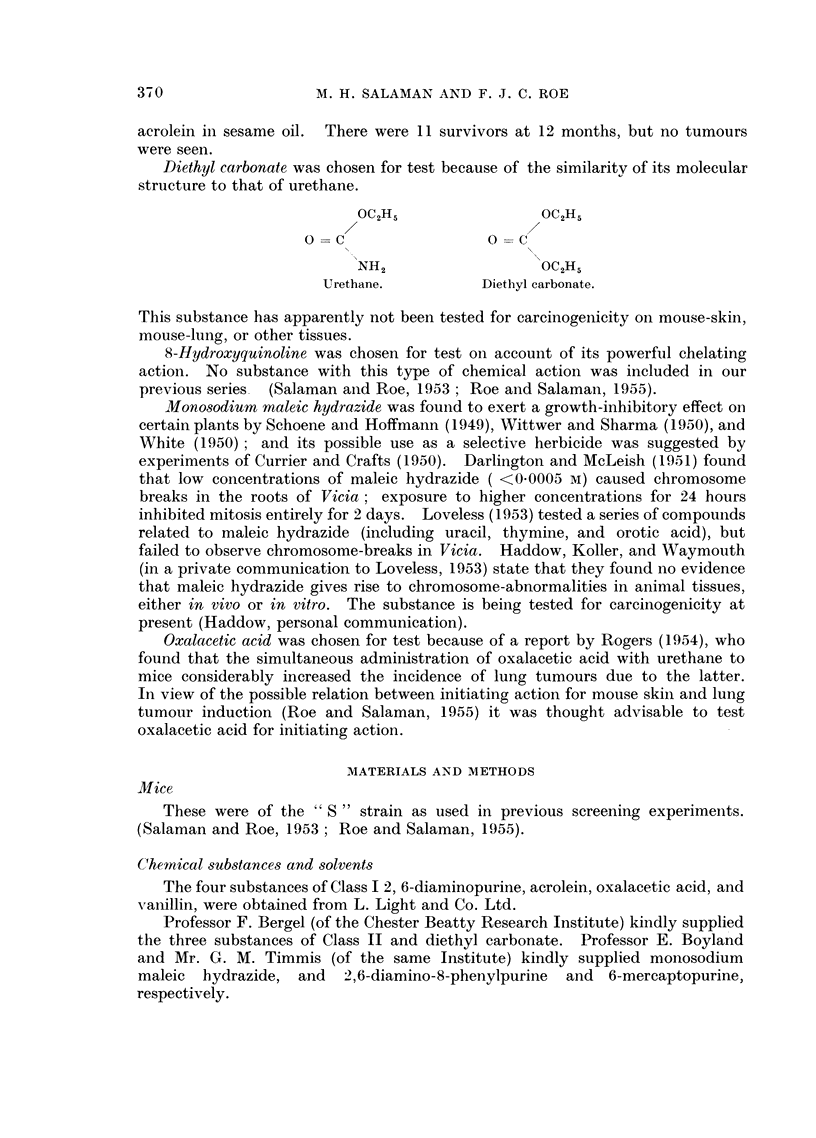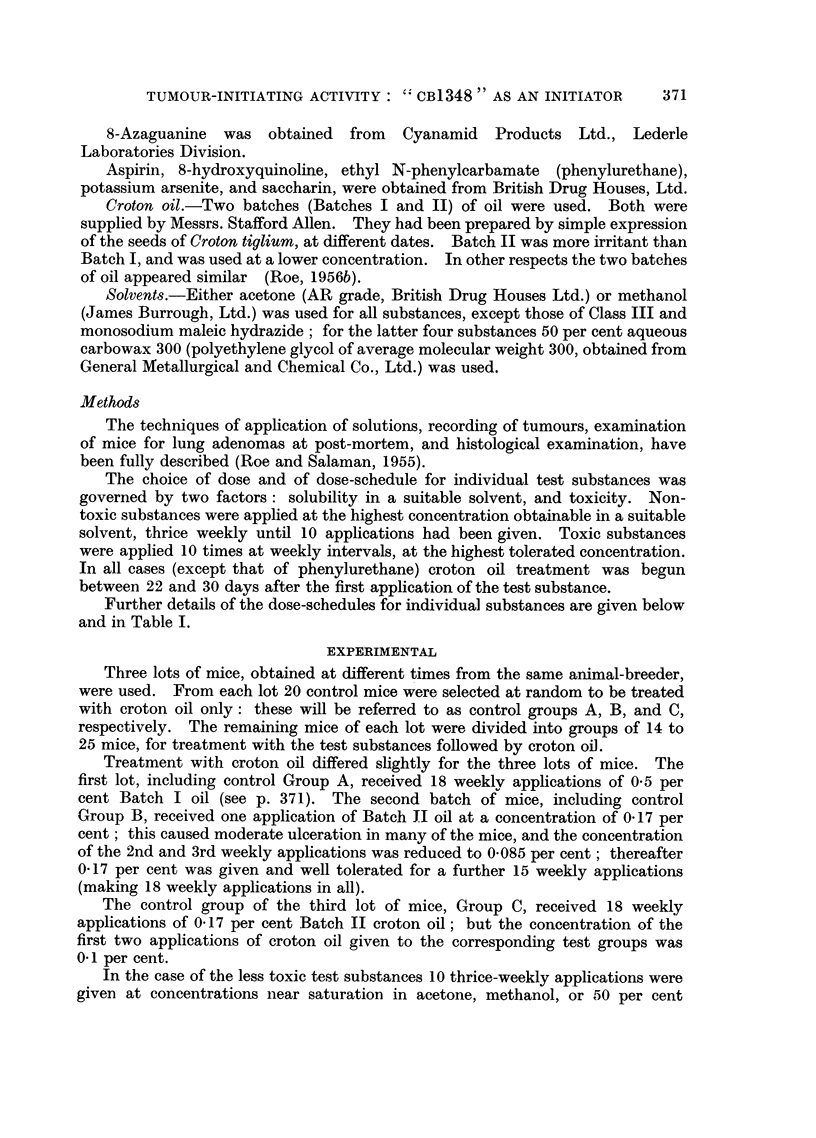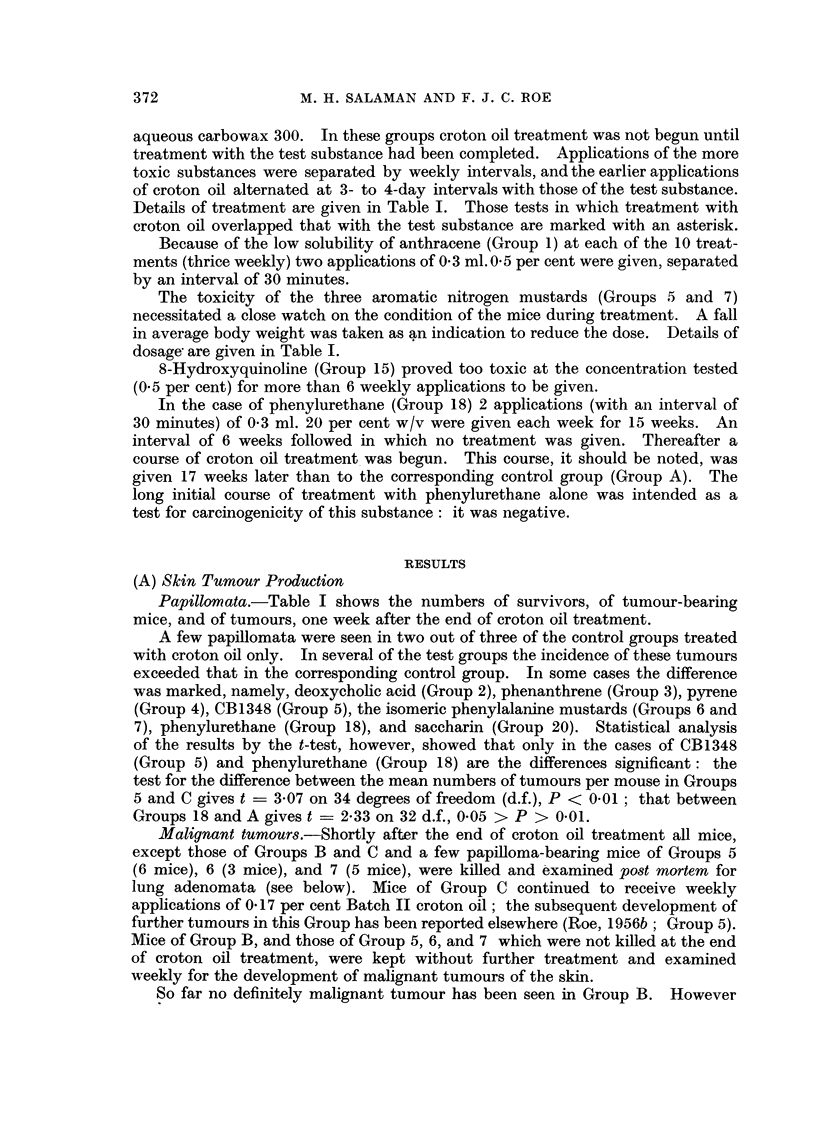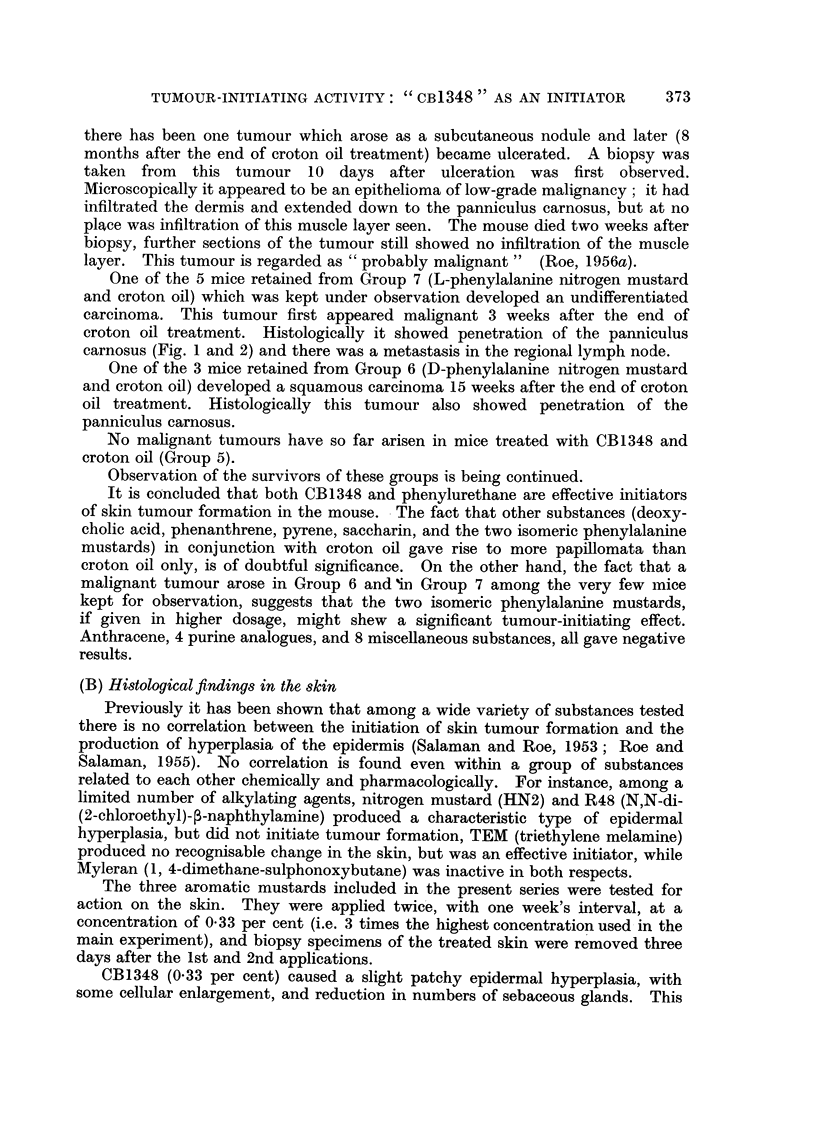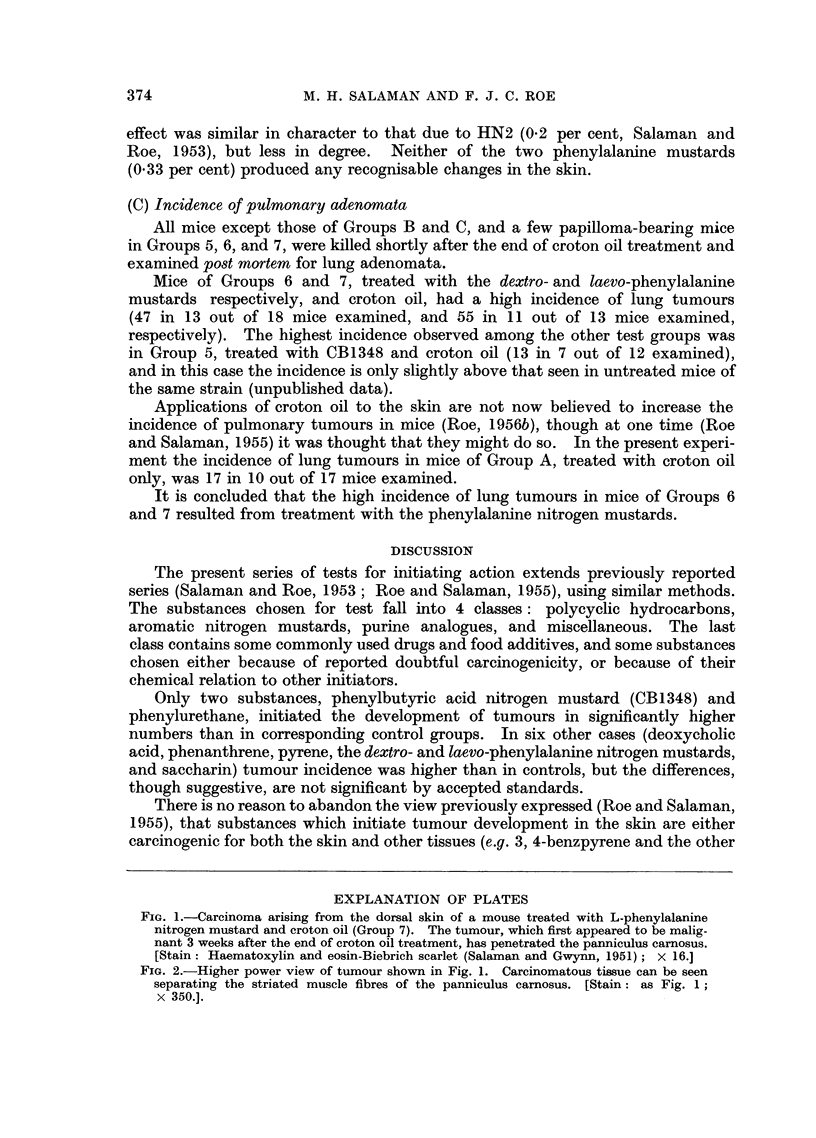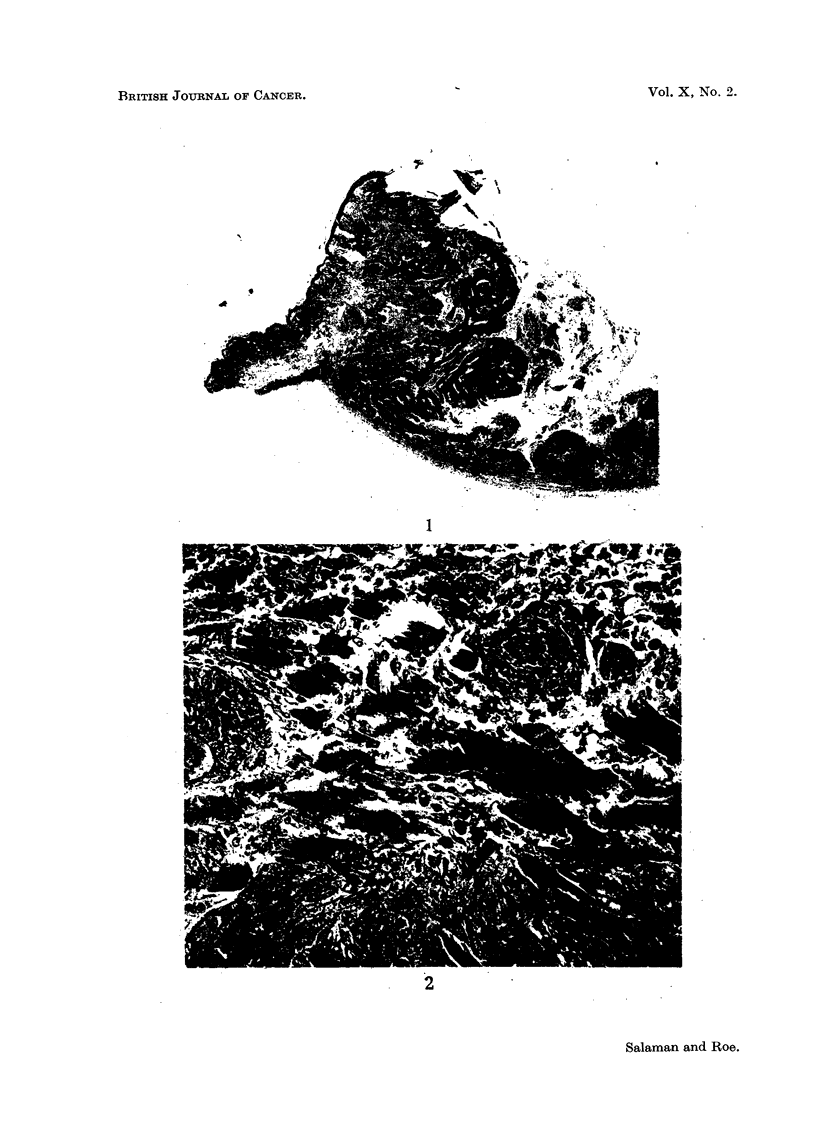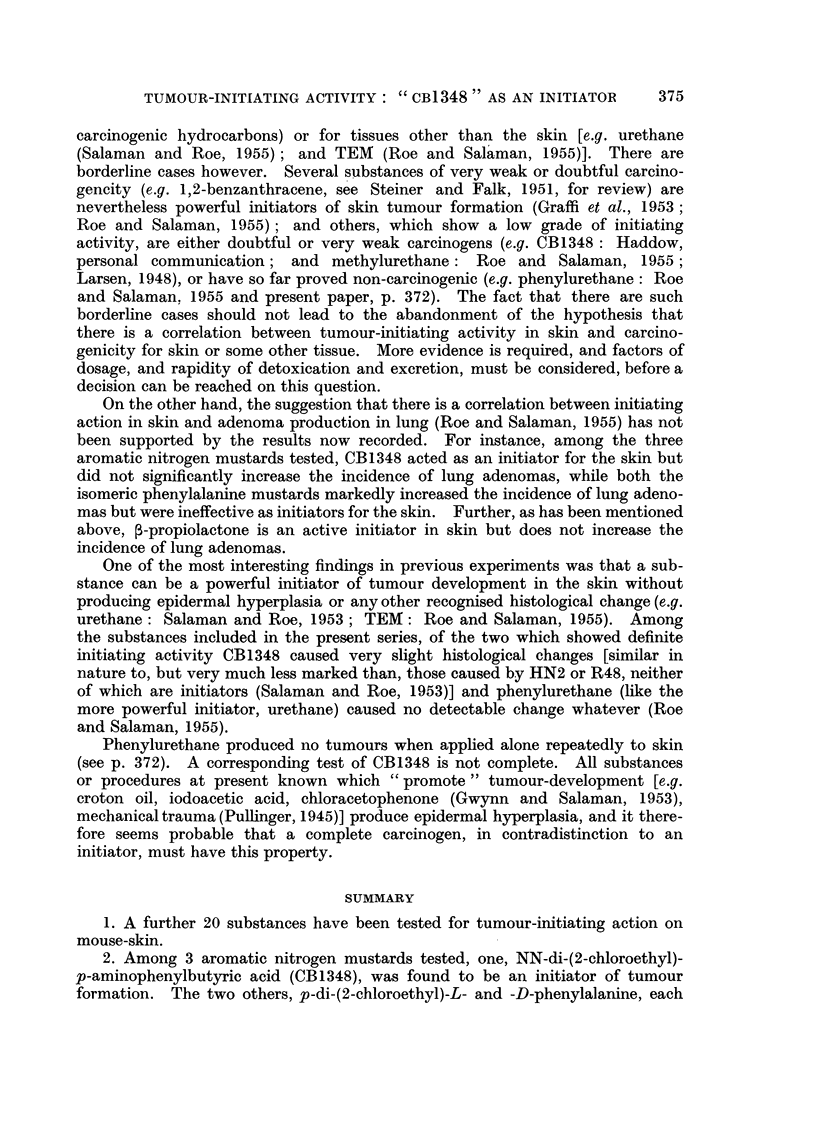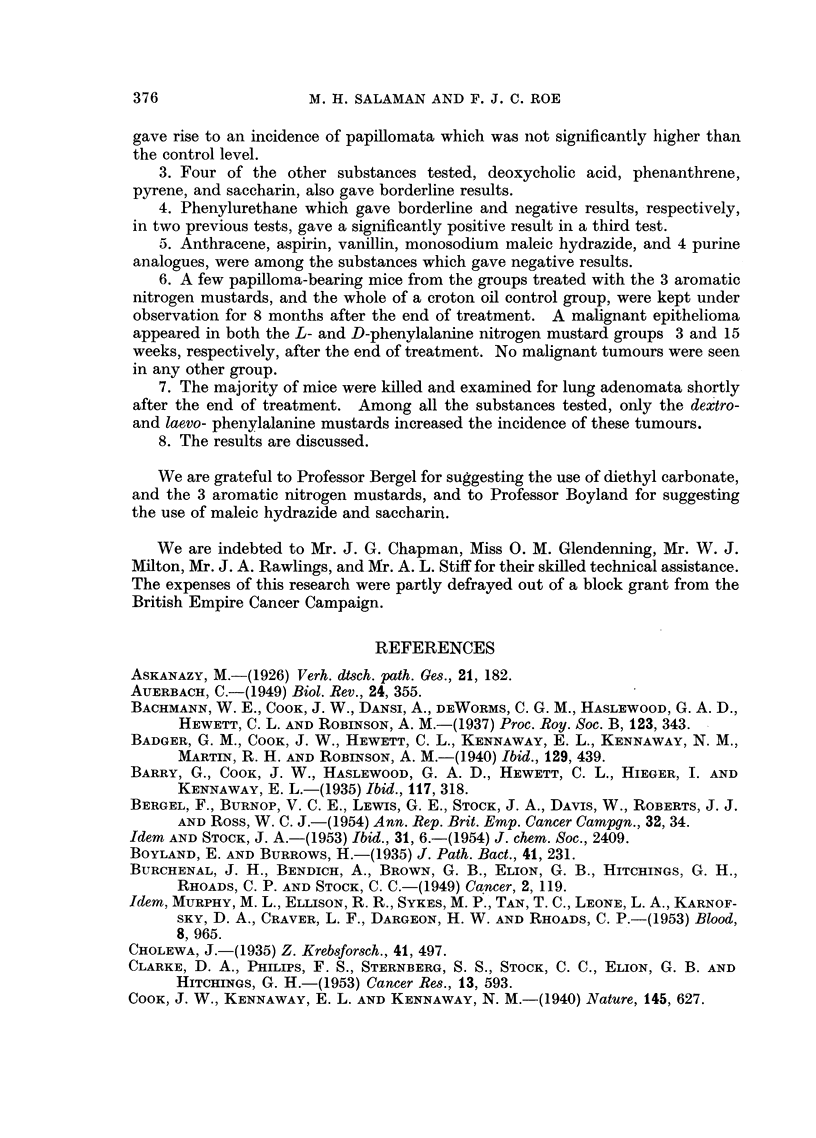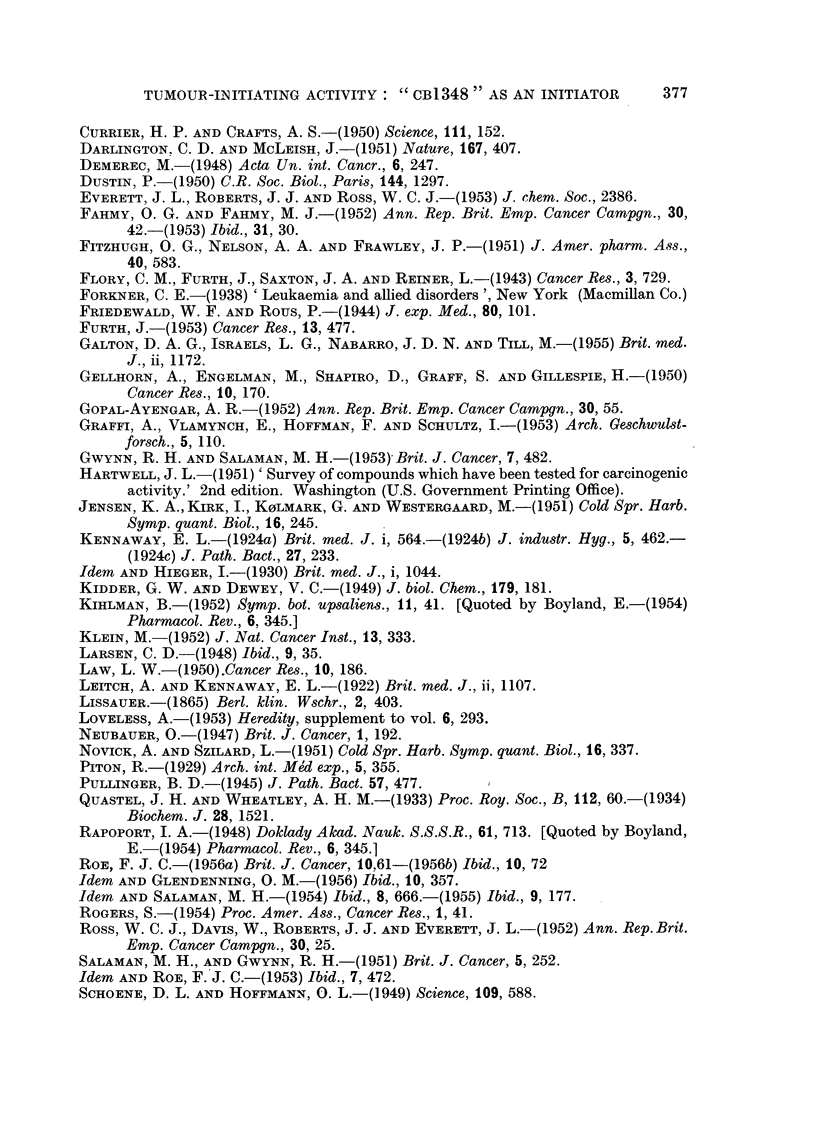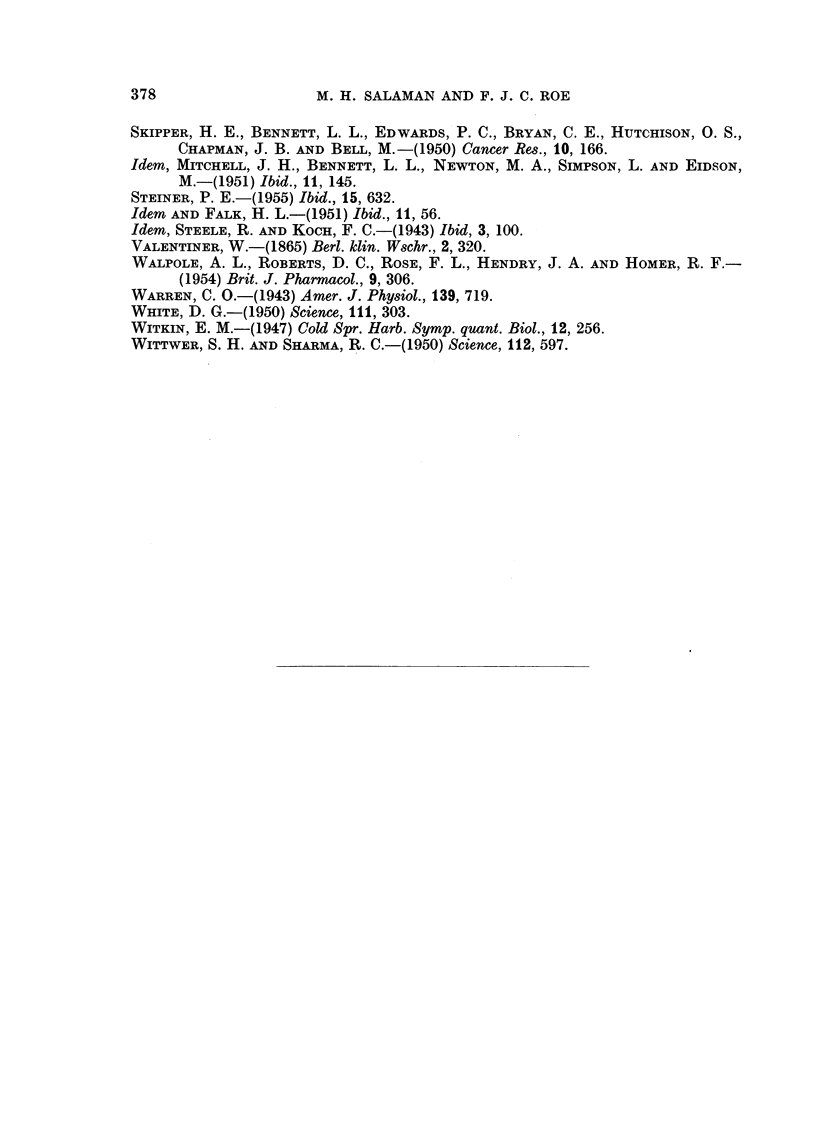# Further Tests for Tumour-Initiating Activity: N,N-DI-(2-Chloroethyl)-P-Aminophenylbutyric Acid (CB1348) as an Initiator of Skin Tumour Formation in the Mouse

**DOI:** 10.1038/bjc.1956.42

**Published:** 1956-06

**Authors:** M. H. Salaman, F. J. C. Roe

## Abstract

**Images:**


### FURTHER TESTS FOR TUMOUR-INITIATING ACTIVITY: N,N-DI-(2-CHLOROETHYL)-*P*-AMINOPHENYLBUTYRIC ACID (CB1348) AS AN INITIATOR OF SKIN TUMOUR FORMATION IN THE MOUSE

M. H. SALAMAN AND F. J. C. ROE

*From the Cancer Research Department, London Hospital Medical College, London, E.*1

Received for publication April 17, 1956.

In previous papers (Salaman and Roe, 1953 ; Roe and Salaman, 1954, 1955), it has been shown that there exist substances which, though not carcinogenic for mouse skin, produce a change in this tissue such that a subsequent short course of croton oil applications, insufficient to produce more than a few tumours in previously untreated skin (Roe, 1956*b*), elicits tumours in considerable numbers. This change was considered to be a form of incomplete carcinogenesis. In accordance with accepted terminologies (Friedewald and Rous, 1944 ; Furth, 1953) such substances were called " tumour-initiators ".

Urethane (ethyl carbamate), 1, 2-benzanthracene, triethylene melamine (TEM), and β-propiolactone have been shown to be tumour-initators for the skin of the mouse. Two derivatives of urethane (ethyl N-phenylcarbamate and ethyl N-methylcarbamate) showed similar activity, but the results required confirmation (Graffi *et al.*, 1953 ; Salaman and Roe, 1953 ; Roe and Salaman, 1955).

In the cases of urethane, and TEM, application to the skin in near-lethal doses without croton oil gave rise to no tumours (nor to any recognisable histological change in the skin) ; and it was concluded that these substances are non-carcinogenic for mouse skin. On the other hand both gave rise to tumours of mouse lung, and TEM has been shown to give rise to sarcomata when injected subcutaneously in the rat (Walpole *et al.*, 1954). 1,2-Benzanthracene is probably very weakly carcinogenic for mouse skin and other tissues (see Steiner and Falk, 1951, for a review of the evidence) ; but in contrast to its weak carcinogenic action, its tumour-initiating action is strong. β-Propiolactone, which had not been tested for carcinogenicity in mouse-skin at the time its tumour-initiating effect was described (Roe and Salaman, 1955), has subsequently been found to be weakly carcinogenic for this tissue (Roe and Glendenning, 1956). It had previously been found to give rise to sarcomata following subcutaneous injection in the rat (Walpole *et al.*, 1954). As in the case of 1,2-benzanthracene, the tumour-initiating action of β-propiolactone in mouse skin is considerably stronger than its carcinogenic action.

It was suggested (Roe and Salaman, 1955) that there might be a relation between tumour-initiating activity for mouse skin and tumour-inducing activity for mouse lung.Ureth ane, TEM, and ethyl N-methylcarbamate possess both activities ; 1,2-benzanthracene definitely possesses the first and possibly the second ; many other substances were shewn to be without either action (including Myleran and colchicine). At the time this suggestion was made it was not known whether β-propiolactone gave rise to tumours in mouse lung. More recently it has been found that intravenous injections of this substance (1 to 10 mg.) failed to increase the incidence of lung tumours in mice (Roe and Glendenning, 1956). It seems therefore that there is no simple correlation between these two activities. There is at present no clear indication of the chemical basis of tumour-initiating action, and no evidence for a definite relation between tumour-initiation and any other biological activity, except perhaps carcinogenicity for other tissues or species (Roe and Salaman, 1955).

The present paper describes the results of tests of a further 20 substances for tumour-initiating action, and a third test of ethyl N-phenylcarbamate (phenyl-urethane), which gave doubtful and negative results respectively on two previous occasions (Roe and Salaman, 1955, pp. 191 and 201). Reasons for the choice of the 20 substances are briefly outlined below.

*Selection of substances*

A list of substances screened is given in Table I. It was found convenient to divide them into four classes : *polycyclic hydrocarbons, aromatic nitrogen mustards, purine analogues,* and *miscellaneous substances.*

In the first class four substances were chosen for test, either because they were thought to be on the borderline between carcinogenicity and non-carcinogenicity, or because they were the parent substances of potent carcinogenic agents. As pointed out above the tumour-initiating action of 1,2-benzanthracene is far stronger than its carcinogenic action, and it was thought possible that the same might be true for other polycyclic hydrocarbons.

*Anthracene* has been tested for carcinogenicity by many workers, but never with a positive result. Kennaway (1924*a*), who pointed out that workmen handling purified anthracene do not suffer from skin lesions, failed to produce skin tumours in mice with this substance (see also Kennaway, 1924*b* ; Kennaway and Hieger, 1930). Barry *et al.* (1935), and later Bachmann *et al.* (1937), painted mice twice weekly with anthracene in benzene for 88 and 104 weeks respectively, and failed to produce tumours. Boyland and Burrows (1935) injected anthracene in colloidal solution subcutaneously into rats, but observed no tumours. Steiner (1955) injected mice subcutaneously with anthracene in tricaprylin, with a similar result. Demerec (1948) stated that anthracene was probably mutagenic for *Drosophila*, but subsequently withdrew this statement (Demerec, 1949, quoted by Auerbach, 1949).

*Deoxycholic acid.*—Occasional epitheliomata have been seen in mice painted with deoxycholic acid repeatedly over long periods (Bachmann *et al.*, 1937 ; Badger *et al.*, 1940 ; Cook, Kennaway and Kennaway, 1940), and subcutaneous injection into mice has given rise to sarcomata after a prolonged period (Badger *et al.*, 1940). Klein (1952) found that deoxycholic acid applied to the skin gave rise to no tumours either when used alone or in conjunction with croton oil. Deoxycholic acid has been found to be mutagenic for bacteria (Witkin, 1947 ; Jensen *et al.*, 1951), and for *Drosophila* by Demerec (1948), but not by Jensen *et al.*, (1951).

*Phenanthrene* has been tested for carcinogenicity both on mouse-skin (Kennaway 1924*a*, 1924*c* ; Kennaway and Hieger, 1930) and also by subcutaneous injection into mice (Steiner, 1955). No tumours have been recorded in any of

TABLE I.—*The Screening of Substances for Tumour-initiating Activity*

| Group. | Number of mice. | Treatment with the test substance. Substance. | Total dose. | Method of application.§ | Treatment with croton oil.† Reference to control group. | Interval between 1st application of test substance and 1st application of croton oil. | Tumour‡ incidence at the end of croton oil treatment. Survivors. | Tumour-bearing mice. | Total tumours |
|---|---|---|---|---|---|---|---|---|---|
| *Class I.—Polycyclic hydrocarbons* | | | | | | | | | |
| 1 | 20 | Anthracene | 30·0 mg. | 20 applications, 0·5% (2, with an interval of 30 minutes, thrice weekly) | As in Group B | 25 days | 17 | 3 | 4 |
| 2 | 20 | Deoxycholic acid | 67·5 mg. | 10 thrice weekly applications, 2·25% in methanol | ,, ,, ,, B | ,, ,, | 20 | 7 | 14 |
| 3 | 20 | Phenanthrene | 0·54 g. | 10 thrice weekly applications, 18·0% | ,, ,, ,, B | ,, ,, | 20 | 5 | 12 |
| 4 | 20 | Pyrene | 0·25 g. | 10 thrice weekly applications 8·3% | ,, ,, ,, B | ,, ,, | 20 | 6 | 9 |
| *Class II.—Aromatic nitrogen mustards* | | | | | | | | | |
| 5 | 25 | NN-di-(2-chloroethyl)-p-aminophenylbutyric acid (CB1348) | 2·7 mg. | 10 weekly applications, 8 at 0·1%, and 2 at 0·05%, in methanol | As in Group C | 30 days* | 19 | 11 | 30 |
| 6 | 25 | *p*-di-(2-chloroethyl)-amino-d-phenylalanine | 3·0 mg. | 10 weekly applications, 0·1% in methanol | ,, ,, ,, C | ,, ,, * | 22 | 4 | 8 |
| 7 | 25 | *p*-di-(2-chloroethyl)-amino-l-phenylalanine | 1·44 mg. | 10 weekly applications, 0·01% to 0·1% in methanol | ,, ,, ,, C | ,, ,, * | 19 | 2 | 7 |

TABLE I—*continued*

| | | | | | | | | | |
|---|---|---|---|---|---|---|---|---|---|
| *Class III.—Purine analogues* | | | | | | | | | |
| 8 | 25 | 8-Azaguanine | 10 mg. | 10 thrice weekly applications, 0·33% in 50% aqueous carbowax 300 | As in Group C | 23 days | 23 | 2 | 4 |
| 9 | 25 | 2,6-Diaminopurine | 10 mg. | 10 thrice weekly applications, 0·33% in 50% aqueous carbowax 300 | ,, ,, ,, C | ,, ,, | 23 | 1 | 1 |
| 10 | 25 | 2,6-Diamino-8-phenyl-purine | 15 mg. | 10 thrice weekly applications, 0·5% in 50% aqueous carbowax 300 | ,, ,, ,, C | ,, ,, | 23 | 2 | 2 |
| 11 | 25 | 6-Mercaptopurine | 10 mg. | 10 thrice weekly applications, 0·33% in 50% aqueous carbowax 300 | ,, ,, ,, C | ,, ,, | 18 | 0 | 0 |
| *Class IV.—Miscellaneous substances* | | | | | | | | | |
| 12 | 20 | Acetylsalicylic acid (aspirin) | 0·54 g. | 10 thrice weekly applications, 18% | As in Group B | 25 days | 19 | 3 | 5 |
| 13 | 15 | Acrolein | 12·6 mg. | 10 weekly applications, 0·5% | ,, ,, ,, B | ,, ,, * | 15 | 2 | 3 |
| 14 | 25 | Diethyl carbonate | 2·9 g. | 10 thrice weekly applications, pure substance | ,, ,, ,, C | 23 ,, | 23 | 2 | 2 |
| 15 | 20 | 8-Hydroxyquinoline | 9·0 mg. | 6 weekly applications, 0·5% | ,, ,, ,, A | 25 ,, * | 14 | 1 | 2 |
| 16 | 25 | Monosodium maleic hydrazide | 0·3 g. | 10 thrice weekly applications, 10%, in 50% aqueous carbowax 300 | ,, ,, ,, C | 23 ,, | 21 | 2 | 2 |
| 17 | 20 | Oxalacetic acid | 160 mg. | 10 thrice weekly applications, 5·3% | ,, ,, ,, B | 22 ,, | 16 | 2 | 3 |
| 18 | 20 | Phenylurethane (ethyl N-phenylcarbamate) | 1·8 g. | 30 applications, 20% (2, with an interval of 30 minutes, each week for 15 weeks) | ,, ,, ,, A | 20 weeks | 17 | 8 | 22 |
| 19 | 14 | Potassium arsenite | 30 mg. | 10 weekly applications, 1% in methanol | ,, ,, ,, B | 25 days* | 14 | 3 | 3 |
| 20 | 20 | Saccharin | 0·24 g. | 10 thrice weekly applications, 8% | ,, ,, ,, B | ,, ,, | 20 | 7 | 14 |
| 21 | 20 | Vanillin | 0·6 g. | 10 thrice weekly applications, 20% | ,, ,, ,, B | ,, ,, | 18 | 4 | 7 |

TABLE I—*continued*

| Group. | Number of mice. | Treatment | | Tumour‡ incidence at the end of croton oil treatment. Survivors. | Tumour-bearing mice. | Total tumours. |
|---|---|---|---|---|---|---|
| *Control groups* | | | | | | |
| A | 20 | Batch I croton oil only | 18 weekly applications, 0·5% | 17 | 1 | 3 |
| B | 20 | Batch II croton oil only | 1 application of 0·17%; 2 of 0·085%, and a further 15 of 0·17%, at weekly intervals throughout | 19 | 4 | 4 |
| C | 20 | Batch II croton oil only | 18 weekly applications, 0·17% | 17 | 0 | 0 |

§ Each application was 0·3 ml. of solution in acetone (unless otherwise stated) at the concentration shown (w/v in the case of solids, and v/v in the case of liquids).

† Croton oil treatment consisted of 18 weekly applications of 0·3 ml. solution in acetone. Appropriate controls for the test groups are indicated in the Table (Groups A, B, and C).

‡ All the tumours recorded below were benign papillomata.

* The earlier weekly applications of croton oil alternated at 3- to 4- day intervals with those of the test substance.

these tests. Graffi *et al.* (1953) applied phenanthrene and croton oil alternately to the backs of mice for a year ; the yield of tumours was not significantly higher than in croton oil controls. Demerec (1948) found phenanthrene to be non-mutagenic for *Drosophila.*

*Pyrene.*—Occasional papillomata have been seen on mouse skin following repeated applications of pyrene by Barry *et al.* (1935), but not by Badger *et al.* (1940). Graffi *et al.* (1953) tested pyrene in the same way as phenanthrene (see previous paragraph) ; the result was again negative. Demerec (1948) found it to be non-mutagenic for *Drosophila.*

The second class consisted of three *aromatic nitrogen mustards.* The first of these, *N,N-di-(2-chloroethyl)-p-aminophenylbutyric acid (CB*1348), was synthesized by Everett, Roberts, and Ross (1953). It was found to have a more powerful tumour-inhibitory effect on the Walker rat carcinoma 256 than several hundred other biological alkylating agents, including a series of closely related mustards. (Ross *et al.*, 1952). Galton *et al.* (1955) reported striking remissions in patients suffering from Hodgkin's disease, lymphocytic lymphoma, chronic lymphocytic leukaemia, and follicular lymphoma, as a result of treatment with CB1348. Haddow (personal communication) injected a group of male rats subcutaneously weekly for 12 weeks with CB1348 in arachis oil. A sarcoma arose at the injection site in one out of 12 rats one year after the last injection. A sarcoma at the injection site was also seen in one of a group of 12 mice similarly treated. Since it is not unknown for sarcomata to arise following injection of arachis oil only (Walpole *et al.*, 1954) the carcinogenicity of CB1348 remains in doubt. CB1348 was found by Fahmy and Fahmy (1952) to be mutagenic for *Drosophila,* and by Gopal-Ayengar (1952) to produce chromosome abnormalities.

The other two substances of this class are the two isomers *p-di-(2-chloroethyl)-amino-L-*, and *D-phenylalanine.* The synthesis of these substances was described by Bergel and Stock (1954). The same authors (1953) reported a remarkable difference in the tumour-inhibitory effects of the two isomers on the Walker rat carcinoma 256, the L-form showing strong inhibition of growth and the D-form only slight activity. Bergel *et al.*, (1954) state that their colleagues, Dr. Elson and Professor Koller, have found similar differences in the effects of the two isomers on the blood picture of the normal rat, and in the production of chromosome abnormalities.

The isomers have not been tested separately for carcinogenicity, but in an experiment (Haddow : personal communication) in which the racemic mixture was injected subcutaneously in arachis oil weekly for 12 weeks into 10 female rats, a sarcoma arose at the injection site in one rat 18 months after the final injection. Two out of 20 mice, similarly treated, developed sarcomata at the injection site. For the reason stated in the case of CB1348 this result is also indecisive. Fahmy and Fahmy (1953 ; and personal communication) found that both the D- and L-forms were mutagenic.

The third class consisted of four *purine analogues.* Three of them have been shown to have tumour-inhibitory or antileukaemic effects : 2,6-diaminopurine (2,6-DAP) by Burchenal *et al.* (1949) and Skipper *et al.* (1950) ; *6-mercaptopurine* (6-MP) by Clarke *et al.* (1953), and Burchenal *et al.* (1953) ; and *8-azaguanine* (8-AG) by Kidder and Dewey (1949), Gellhorn *et al.* (1950), and Law (1950). It will be recalled that two known tumour-initiators, urethane and TEM (Roe and Salaman, 1955), also possess these properties. 2,6-DAP and 6-MP have both been shown to induce chromosome abnormalities, the former in mice by Dustin (1950), and the latter in *Allium* and *Pisum* by Kihlman (1952). 8-AG was found by Novick and Szilard (1951) to be mutagenic for *E. coli*.

2,6-*Diamino*-8-*phenylpurine*, a purine analogue recently synthesized by Mr. G. M. Timmis of the Chester Beatty Research Institute, was also tested. Little is known of its biological activity at present.

The fourth class consisted of 10 *miscellaneous* substances, which were chosen for test for a variety of reasons.

*Phenylurethane*, chemically and pharmacologically related to urethane, had been tested twice before, with a doubtful and a negative result respectively (Roe and Salaman, 1955). The properties of this substance were discussed in that paper. In those tests 0·5 g. and 1·32 g., respectively, were applied. In the test described below a total of 1·8 g. was applied.

*Potassium arsenite*, the active constituent of Fowler's solution, was selected because of its antileukaemic action. It was first used in the treatment of leukaemia by Valentiner (1865), and Lissauer (1865), but went out of fashion with the introduction of radiotherapy. Forkner (1938) reintroduced it, and Flory *et al.* (1943) found it efficacious in several strains of mouse leukaemia. Skipper *et al.* (1951) found that potassium arsenite, like urethane, nitrogen mustard, folic acid antagonists, and certain purine analogues (including 2,6-DAP and 8-AG), inhibited the incorporation of labelled formate in the synthesis of nucleic acid purines. Warren (1943) found that arsenite depressed respiration and, probably as a secondary effect, accelerated aerobic glycolysis (urethane also inhibits aerobic glycolysis : Quastel and Wheatley, 1933, 1934). Piton (1929) found that potassium arsenite stimulated mitosis in the intestinal epithelium of the mouse, but that this was followed by death of the mitotic cells. Leitch and Kennaway (1922) painted mice thrice-weekly with a 0·12 per cent solution of potassium arsenite in ethanol, and observed an epithelioma and a benign wart in 1 out of the 30 mice which survived for 3 months or more. The epithelioma had metastasised to the lung. Askanazy (1926) records the development of stomach neoplasms in 4 rats after prolonged administration of potassium arsenite in the drinking water. Cholewa (1935) observed lung tumours in mice given potassium arsenite by mouth, and a sarcoma of the ear in one of two rabbits injected subcutaneously with the substance. Evidence for the carcinogenicity of potassium arsenite, and other arsenical compounds, for both man and experimental animals, is reviewed and discussed by Neubauer (1947).

Three substances, *aspirin*, *saccharin*, and *vanillin*, were chosen for no other reason than that they are commonly ingested. Hartwell (1951) lists four experiments in which aspirin was tested for carcinogenicity in rats ; in three the substance was given by mouth, and in one subcutaneously in lard. No tumours were seen in any of these experiments. Fitzhugh, Nelson, and Frawley (1951) reported an increased incidence of abdominal lymphosarcomata in rats fed for 102 weeks or more on a diet contain 5 per cent saccharin, but the number of animals involved was small (4 with abdominal lymphosarcomata among 18 rats which survived for 1 year or more). Vanillin has apparently not been tested for carcinogenicity.

*Acrolein*, an unsaturated aldehyde ($CH_2 = CH.CHO$), was chosen mainly because it is actively mutagenic for *Drosophila* (Rapoport, 1948). Steiner, Steele, and Koch (1943) injected 15 mice subcutaneously weekly for 24 weeks with acrolein in sesame oil. There were 11 survivors at 12 months, but no tumours were seen.

*Diethyl carbonate* was chosen for test because of the similarity of its molecular structure to that of urethane.

Urethane.

Diethyl carbonate.

This substance has apparently not been tested for carcinogenicity on mouse-skin, mouse-lung, or other tissues.

8-*Hydroxyquinoline* was chosen for test on account of its powerful chelating action. No substance with this type of chemical action was included in our previous series (Salaman and Roe, 1953 ; Roe and Salaman, 1955).

*Monosodium maleic hydrazide* was found to exert a growth-inhibitory effect on certain plants by Schoene and Hoffmann (1949), Wittwer and Sharma (1950), and White (1950) ; and its possible use as a selective herbicide was suggested by experiments of Currier and Crafts (1950). Darlington and McLeish (1951) found that low concentrations of maleic hydrazide ( $<0{\cdot}0005$ M) caused chromosome breaks in the roots of *Vicia* ; exposure to higher concentrations for 24 hours inhibited mitosis entirely for 2 days. Loveless (1953) tested a series of compounds related to maleic hydrazide (including uracil, thymine, and orotic acid), but failed to observe chromosome-breaks in *Vicia.* Haddow, Koller, and Waymouth (in a private communication to Loveless, 1953) state that they found no evidence that maleic hydrazide gives rise to chromosome-abnormalities in animal tissues, either *in vivo* or *in vitro.* The substance is being tested for carcinogenicity at present (Haddow, personal communication).

*Oxalacetic acid* was chosen for test because of a report by Rogers (1954), who found that the simultaneous administration of oxalacetic acid with urethane to mice considerably increased the incidence of lung tumours due to the latter. In view of the possible relation between initiating action for mouse skin and lung tumour induction (Roe and Salaman, 1955) it was thought advisable to test oxalacetic acid for initiating action.

### MATERIALS AND METHODS

### *Mice*

These were of the "S" strain as used in previous screening experiments. (Salaman and Roe, 1953 ; Roe and Salaman, 1955).

### *Chemical substances and solvents*

The four substances of Class I 2, 6-diaminopurine, acrolein, oxalacetic acid, and vanillin, were obtained from L. Light and Co. Ltd.

Professor F. Bergel (of the Chester Beatty Research Institute) kindly supplied the three substances of Class II and diethyl carbonate. Professor E. Boyland and Mr. G. M. Timmis (of the same Institute) kindly supplied monosodium maleic hydrazide, and 2,6-diamino-8-phenylpurine and 6-mercaptopurine, respectively.

8-Azaguanine was obtained from Cyanamid Products Ltd., Lederle Laboratories Division.

Aspirin, 8-hydroxyquinoline, ethyl N-phenylcarbamate (phenylurethane), potassium arsenite, and saccharin, were obtained from British Drug Houses, Ltd.

*Croton oil.*—Two batches (Batches I and II) of oil were used. Both were supplied by Messrs. Stafford Allen. They had been prepared by simple expression of the seeds of *Croton tiglium*, at different dates. Batch II was more irritant than Batch I, and was used at a lower concentration. In other respects the two batches of oil appeared similar (Roe, 1956*b*).

*Solvents.*—Either acetone (AR grade, British Drug Houses Ltd.) or methanol (James Burrough, Ltd.) was used for all substances, except those of Class III and monosodium maleic hydrazide ; for the latter four substances 50 per cent aqueous carbowax 300 (polyethylene glycol of average molecular weight 300, obtained from General Metallurgical and Chemical Co., Ltd.) was used.

*Methods*

The techniques of application of solutions, recording of tumours, examination of mice for lung adenomas at post-mortem, and histological examination, have been fully described (Roe and Salaman, 1955).

The choice of dose and of dose-schedule for individual test substances was governed by two factors : solubility in a suitable solvent, and toxicity. Non-toxic substances were applied at the highest concentration obtainable in a suitable solvent, thrice weekly until 10 applications had been given. Toxic substances were applied 10 times at weekly intervals, at the highest tolerated concentration. In all cases (except that of phenylurethane) croton oil treatment was begun between 22 and 30 days after the first application of the test substance.

Further details of the dose-schedules for individual substances are given below and in Table I.

EXPERIMENTAL

Three lots of mice, obtained at different times from the same animal-breeder, were used. From each lot 20 control mice were selected at random to be treated with croton oil only : these will be referred to as control groups A, B, and C, respectively. The remaining mice of each lot were divided into groups of 14 to 25 mice, for treatment with the test substances followed by croton oil.

Treatment with croton oil differed slightly for the three lots of mice. The first lot, including control Group A, received 18 weekly applications of 0·5 per cent Batch I oil (see p. 371). The second batch of mice, including control Group B, received one application of Batch II oil at a concentration of 0·17 per cent ; this caused moderate ulceration in many of the mice, and the concentration of the 2nd and 3rd weekly applications was reduced to 0·085 per cent ; thereafter 0·17 per cent was given and well tolerated for a further 15 weekly applications (making 18 weekly applications in all).

The control group of the third lot of mice, Group C, received 18 weekly applications of 0·17 per cent Batch II croton oil ; but the concentration of the first two applications of croton oil given to the corresponding test groups was 0·1 per cent.

In the case of the less toxic test substances 10 thrice-weekly applications were given at concentrations near saturation in acetone, methanol, or 50 per cent aqueous carbowax 300. In these groups croton oil treatment was not begun until treatment with the test substance had been completed. Applications of the more toxic substances were separated by weekly intervals, and the earlier applications of croton oil alternated at 3- to 4-day intervals with those of the test substance. Details of treatment are given in Table I. Those tests in which treatment with croton oil overlapped that with the test substance are marked with an asterisk.

Because of the low solubility of anthracene (Group 1) at each of the 10 treatments (thrice weekly) two applications of 0·3 ml. 0·5 per cent were given, separated by an interval of 30 minutes.

The toxicity of the three aromatic nitrogen mustards (Groups 5 and 7) necessitated a close watch on the condition of the mice during treatment. A fall in average body weight was taken as an indication to reduce the dose. Details of dosage are given in Table I.

8-Hydroxyquinoline (Group 15) proved too toxic at the concentration tested (0·5 per cent) for more than 6 weekly applications to be given.

In the case of phenylurethane (Group 18) 2 applications (with an interval of 30 minutes) of 0·3 ml. 20 per cent w/v were given each week for 15 weeks. An interval of 6 weeks followed in which no treatment was given. Thereafter a course of croton oil treatment was begun. This course, it should be noted, was given 17 weeks later than to the corresponding control group (Group A). The long initial course of treatment with phenylurethane alone was intended as a test for carcinogenicity of this substance : it was negative.

### RESULTS

### (A) *Skin Tumour Production*

*Papillomata.*—Table I shows the numbers of survivors, of tumour-bearing mice, and of tumours, one week after the end of croton oil treatment.

A few papillomata were seen in two out of three of the control groups treated with croton oil only. In several of the test groups the incidence of these tumours exceeded that in the corresponding control group. In some cases the difference was marked, namely, deoxycholic acid (Group 2), phenanthrene (Group 3), pyrene (Group 4), CB1348 (Group 5), the isomeric phenylalanine mustards (Groups 6 and 7), phenylurethane (Group 18), and saccharin (Group 20). Statistical analysis of the results by the *t*-test, however, showed that only in the cases of CB1348 (Group 5) and phenylurethane (Group 18) are the differences significant : the test for the difference between the mean numbers of tumours per mouse in Groups 5 and C gives $t = 3·07$ on 34 degrees of freedom (d.f.), $P < 0·01$ ; that between Groups 18 and A gives $t = 2·33$ on 32 d.f., $0·05 > P > 0·01$.

*Malignant tumours.*—Shortly after the end of croton oil treatment all mice, except those of Groups B and C and a few papilloma-bearing mice of Groups 5 (6 mice), 6 (3 mice), and 7 (5 mice), were killed and examined *post mortem* for lung adenomata (see below). Mice of Group C continued to receive weekly applications of 0·17 per cent Batch II croton oil ; the subsequent development of further tumours in this Group has been reported elsewhere (Roe, 1956*b* ; Group 5). Mice of Group B, and those of Group 5, 6, and 7 which were not killed at the end of croton oil treatment, were kept without further treatment and examined weekly for the development of malignant tumours of the skin.

So far no definitely malignant tumour has been seen in Group B. However there has been one tumour which arose as a subcutaneous nodule and later (8 months after the end of croton oil treatment) became ulcerated. A biopsy was taken from this tumour 10 days after ulceration was first observed. Microscopically it appeared to be an epithelioma of low-grade malignancy; it had infiltrated the dermis and extended down to the panniculus carnosus, but at no place was infiltration of this muscle layer seen. The mouse died two weeks after biopsy, further sections of the tumour still showed no infiltration of the muscle layer. This tumour is regarded as "probably malignant" (Roe, 1956*a*).

One of the 5 mice retained from Group 7 (L-phenylalanine nitrogen mustard and croton oil) which was kept under observation developed an undifferentiated carcinoma. This tumour first appeared malignant 3 weeks after the end of croton oil treatment. Histologically it showed penetration of the panniculus carnosus (Fig. 1 and 2) and there was a metastasis in the regional lymph node.

One of the 3 mice retained from Group 6 (D-phenylalanine nitrogen mustard and croton oil) developed a squamous carcinoma 15 weeks after the end of croton oil treatment. Histologically this tumour also showed penetration of the panniculus carnosus.

No malignant tumours have so far arisen in mice treated with CB1348 and croton oil (Group 5).

Observation of the survivors of these groups is being continued.

It is concluded that both CB1348 and phenylurethane are effective initiators of skin tumour formation in the mouse. The fact that other substances (deoxycholic acid, phenanthrene, pyrene, saccharin, and the two isomeric phenylalanine mustards) in conjunction with croton oil gave rise to more papillomata than croton oil only, is of doubtful significance. On the other hand, the fact that a malignant tumour arose in Group 6 and in Group 7 among the very few mice kept for observation, suggests that the two isomeric phenylalanine mustards, if given in higher dosage, might shew a significant tumour-initiating effect. Anthracene, 4 purine analogues, and 8 miscellaneous substances, all gave negative results.

(B) *Histological findings in the skin*

Previously it has been shown that among a wide variety of substances tested there is no correlation between the initiation of skin tumour formation and the production of hyperplasia of the epidermis (Salaman and Roe, 1953; Roe and Salaman, 1955). No correlation is found even within a group of substances related to each other chemically and pharmacologically. For instance, among a limited number of alkylating agents, nitrogen mustard (HN2) and R48 (N,N-di-(2-chloroethyl)-β-naphthylamine) produced a characteristic type of epidermal hyperplasia, but did not initiate tumour formation, TEM (triethylene melamine) produced no recognisable change in the skin, but was an effective initiator, while Myleran (1, 4-dimethane-sulphonoxybutane) was inactive in both respects.

The three aromatic mustards included in the present series were tested for action on the skin. They were applied twice, with one week's interval, at a concentration of 0·33 per cent (i.e. 3 times the highest concentration used in the main experiment), and biopsy specimens of the treated skin were removed three days after the 1st and 2nd applications.

CB1348 (0·33 per cent) caused a slight patchy epidermal hyperplasia, with some cellular enlargement, and reduction in numbers of sebaceous glands. This effect was similar in character to that due to HN2 (0·2 per cent, Salaman and Roe, 1953), but less in degree. Neither of the two phenylalanine mustards (0·33 per cent) produced any recognisable changes in the skin.

(C) *Incidence of pulmonary adenomata*

All mice except those of Groups B and C, and a few papilloma-bearing mice in Groups 5, 6, and 7, were killed shortly after the end of croton oil treatment and examined *post mortem* for lung adenomata.

Mice of Groups 6 and 7, treated with the *dextro-* and *laevo-*phenylalanine mustards respectively, and croton oil, had a high incidence of lung tumours (47 in 13 out of 18 mice examined, and 55 in 11 out of 13 mice examined, respectively). The highest incidence observed among the other test groups was in Group 5, treated with CB1348 and croton oil (13 in 7 out of 12 examined), and in this case the incidence is only slightly above that seen in untreated mice of the same strain (unpublished data).

Applications of croton oil to the skin are not now believed to increase the incidence of pulmonary tumours in mice (Roe, 1956*b*), though at one time (Roe and Salaman, 1955) it was thought that they might do so. In the present experiment the incidence of lung tumours in mice of Group A, treated with croton oil only, was 17 in 10 out of 17 mice examined.

It is concluded that the high incidence of lung tumours in mice of Groups 6 and 7 resulted from treatment with the phenylalanine nitrogen mustards.

### DISCUSSION

The present series of tests for initiating action extends previously reported series (Salaman and Roe, 1953 ; Roe and Salaman, 1955), using similar methods. The substances chosen for test fall into 4 classes : polycyclic hydrocarbons, aromatic nitrogen mustards, purine analogues, and miscellaneous. The last class contains some commonly used drugs and food additives, and some substances chosen either because of reported doubtful carcinogenicity, or because of their chemical relation to other initiators.

Only two substances, phenylbutyric acid nitrogen mustard (CB1348) and phenylurethane, initiated the development of tumours in significantly higher numbers than in corresponding control groups. In six other cases (deoxycholic acid, phenanthrene, pyrene, the *dextro-* and *laevo-*phenylalanine nitrogen mustards, and saccharin) tumour incidence was higher than in controls, but the differences, though suggestive, are not significant by accepted standards.

There is no reason to abandon the view previously expressed (Roe and Salaman, 1955), that substances which initiate tumour development in the skin are either carcinogenic for both the skin and other tissues (*e.g.* 3, 4-benzpyrene and the other

---

### EXPLANATION OF PLATES

FIG. 1.—Carcinoma arising from the dorsal skin of a mouse treated with L-phenylalanine nitrogen mustard and croton oil (Group 7). The tumour, which first appeared to be malignant 3 weeks after the end of croton oil treatment, has penetrated the panniculus carnosus. [Stain : Haematoxylin and eosin-Biebrich scarlet (Salaman and Gwynn, 1951) ; × 16.]

FIG. 2.—Higher power view of tumour shown in Fig. 1. Carcinomatous tissue can be seen separating the striated muscle fibres of the panniculus carnosus. [Stain : as Fig. 1 ; × 350.].

1

2

carcinogenic hydrocarbons) or for tissues other than the skin [*e.g.* urethane (Salaman and Roe, 1955) ; and TEM (Roe and Salaman, 1955)]. There are borderline cases however. Several substances of very weak or doubtful carcinogencity (*e.g.* 1,2-benzanthracene, see Steiner and Falk, 1951, for review) are nevertheless powerful initiators of skin tumour formation (Graffi *et al.*, 1953 ; Roe and Salaman, 1955) ; and others, which show a low grade of initiating activity, are either doubtful or very weak carcinogens (*e.g.* CB1348 : Haddow, personal communication ; and methylurethane : Roe and Salaman, 1955 ; Larsen, 1948), or have so far proved non-carcinogenic (*e.g.* phenylurethane : Roe and Salaman, 1955 and present paper, p. 372). The fact that there are such borderline cases should not lead to the abandonment of the hypothesis that there is a correlation between tumour-initiating activity in skin and carcinogenicity for skin or some other tissue. More evidence is required, and factors of dosage, and rapidity of detoxication and excretion, must be considered, before a decision can be reached on this question.

On the other hand, the suggestion that there is a correlation between initiating action in skin and adenoma production in lung (Roe and Salaman, 1955) has not been supported by the results now recorded. For instance, among the three aromatic nitrogen mustards tested, CB1348 acted as an initiator for the skin but did not significantly increase the incidence of lung adenomas, while both the isomeric phenylalanine mustards markedly increased the incidence of lung adenomas but were ineffective as initiators for the skin. Further, as has been mentioned above, β-propiolactone is an active initiator in skin but does not increase the incidence of lung adenomas.

One of the most interesting findings in previous experiments was that a substance can be a powerful initiator of tumour development in the skin without producing epidermal hyperplasia or any other recognised histological change (*e.g.* urethane : Salaman and Roe, 1953 ; TEM : Roe and Salaman, 1955). Among the substances included in the present series, of the two which showed definite initiating activity CB1348 caused very slight histological changes [similar in nature to, but very much less marked than, those caused by HN2 or R48, neither of which are initiators (Salaman and Roe, 1953)] and phenylurethane (like the more powerful initiator, urethane) caused no detectable change whatever (Roe and Salaman, 1955).

Phenylurethane produced no tumours when applied alone repeatedly to skin (see p. 372). A corresponding test of CB1348 is not complete. All substances or procedures at present known which "promote" tumour-development [*e.g.* croton oil, iodoacetic acid, chloracetophenone (Gwynn and Salaman, 1953), mechanical trauma (Pullinger, 1945)] produce epidermal hyperplasia, and it therefore seems probable that a complete carcinogen, in contradistinction to an initiator, must have this property.

### SUMMARY

1. A further 20 substances have been tested for tumour-initiating action on mouse-skin.

2. Among 3 aromatic nitrogen mustards tested, one, NN-di-(2-chloroethyl)-*p*-aminophenylbutyric acid (CB1348), was found to be an initiator of tumour formation. The two others, *p*-di-(2-chloroethyl)-*L*- and -*D*-phenylalanine, each gave rise to an incidence of papillomata which was not significantly higher than the control level.

3. Four of the other substances tested, deoxycholic acid, phenanthrene, pyrene, and saccharin, also gave borderline results.

4. Phenylurethane which gave borderline and negative results, respectively, in two previous tests, gave a significantly positive result in a third test.

5. Anthracene, aspirin, vanillin, monosodium maleic hydrazide, and 4 purine analogues, were among the substances which gave negative results.

6. A few papilloma-bearing mice from the groups treated with the 3 aromatic nitrogen mustards, and the whole of a croton oil control group, were kept under observation for 8 months after the end of treatment. A malignant epithelioma appeared in both the *L*- and *D*-phenylalanine nitrogen mustard groups 3 and 15 weeks, respectively, after the end of treatment. No malignant tumours were seen in any other group.

7. The majority of mice were killed and examined for lung adenomata shortly after the end of treatment. Among all the substances tested, only the *dextro*- and *laevo*- phenylalanine mustards increased the incidence of these tumours.

8. The results are discussed.

We are grateful to Professor Bergel for suggesting the use of diethyl carbonate, and the 3 aromatic nitrogen mustards, and to Professor Boyland for suggesting the use of maleic hydrazide and saccharin.

We are indebted to Mr. J. G. Chapman, Miss O. M. Glendenning, Mr. W. J. Milton, Mr. J. A. Rawlings, and Mr. A. L. Stiff for their skilled technical assistance. The expenses of this research were partly defrayed out of a block grant from the British Empire Cancer Campaign.

### REFERENCES

Askanazy, M.—(1926) *Verh. dtsch. path. Ges.*, **21**, 182.

Auerbach, C.—(1949) *Biol. Rev.*, **24**, 355.

Bachmann, W. E., Cook, J. W., Dansi, A., deWorms, C. G. M., Haslewood, G. A. D., Hewett, C. L. and Robinson, A. M.—(1937) *Proc. Roy. Soc.* B, **123**, 343.

Badger, G. M., Cook, J. W., Hewett, C. L., Kennaway, E. L., Kennaway, N. M., Martin, R. H. and Robinson, A. M.—(1940) *Ibid.*, **129**, 439.

Barry, G., Cook, J. W., Haslewood, G. A. D., Hewett, C. L., Hieger, I. and Kennaway, E. L.—(1935) *Ibid.*, **117**, 318.

Bergel, F., Burnop, V. C. E., Lewis, G. E., Stock, J. A., Davis, W., Roberts, J. J. and Ross, W. C. J.—(1954) *Ann. Rep. Brit. Emp. Cancer Campgn.*, **32**, 34.

*Idem* and Stock, J. A.—(1953) *Ibid.*, **31**, 6.—(1954) *J. chem. Soc.*, 2409.

Boyland, E. and Burrows, H.—(1935) *J. Path. Bact.*, **41**, 231.

Burchenal, J. H., Bendich, A., Brown, G. B., Elion, G. B., Hitchings, G. H., Rhoads, C. P. and Stock, C. C.—(1949) *Cancer*, **2**, 119.

*Idem*, Murphy, M. L., Ellison, R. R., Sykes, M. P., Tan, T. C., Leone, L. A., Karnofsky, D. A., Craver, L. F., Dargeon, H. W. and Rhoads, C. P.—(1953) *Blood*, **8**, 965.

Cholewa, J.—(1935) *Z. Krebsforsch.*, **41**, 497.

Clarke, D. A., Philips, F. S., Sternberg, S. S., Stock, C. C., Elion, G. B. and Hitchings, G. H.—(1953) *Cancer Res.*, **13**, 593.

Cook, J. W., Kennaway, E. L. and Kennaway, N. M.—(1940) *Nature*, **145**, 627.

CURRIER, H. P. AND CRAFTS, A. S.—(1950) *Science*, **111**, 152.
DARLINGTON, C. D. AND McLEISH, J.—(1951) *Nature*, **167**, 407.
DEMEREC, M.—(1948) *Acta Un. int. Cancr.*, **6**, 247.
DUSTIN, P.—(1950) *C.R. Soc. Biol., Paris*, **144**, 1297.
EVERETT, J. L., ROBERTS, J. J. AND ROSS, W. C. J.—(1953) *J. chem. Soc.*, 2386.
FAHMY, O. G. AND FAHMY, M. J.—(1952) *Ann. Rep. Brit. Emp. Cancer Campgn.*, **30**, 42.—(1953) *Ibid.*, **31**, 30.
FITZHUGH, O. G., NELSON, A. A. AND FRAWLEY, J. P.—(1951) *J. Amer. pharm. Ass.*, **40**, 583.
FLORY, C. M., FURTH, J., SAXTON, J. A. AND REINER, L.—(1943) *Cancer Res.*, **3**, 729.
FORKNER, C. E.—(1938) ' Leukaemia and allied disorders ', New York (Macmillan Co.)
FRIEDEWALD, W. F. AND ROUS, P.—(1944) *J. exp. Med.*, **80**, 101.
FURTH, J.—(1953) *Cancer Res.*, **13**, 477.
GALTON, D. A. G., ISRAELS, L. G., NABARRO, J. D. N. AND TILL, M.—(1955) *Brit. med. J.*, ii, 1172.
GELLHORN, A., ENGELMAN, M., SHAPIRO, D., GRAFF, S. AND GILLESPIE, H.—(1950) *Cancer Res.*, **10**, 170.
GOPAL-AYENGAR, A. R.—(1952) *Ann. Rep. Brit. Emp. Cancer Campgn.*, **30**, 55.
GRAFFI, A., VLAMYNCH, E., HOFFMAN, F. AND SCHULTZ, I.—(1953) *Arch. Geschwulstforsch.*, **5**, 110.
GWYNN, R. H. AND SALAMAN, M. H.—(1953) *Brit. J. Cancer*, **7**, 482.
HARTWELL, J. L.—(1951) ' Survey of compounds which have been tested for carcinogenic activity.' 2nd edition. Washington (U.S. Government Printing Office).
JENSEN, K. A., KIRK, I., KØLMARK, G. AND WESTERGAARD, M.—(1951) *Cold Spr. Harb. Symp. quant. Biol.*, **16**, 245.
KENNAWAY, E. L.—(1924*a*) *Brit. med. J.* i, 564.—(1924*b*) *J. industr. Hyg.*, **5**, 462.—(1924*c*) *J. Path. Bact.*, **27**, 233.
*Idem* AND HIEGER, I.—(1930) *Brit. med. J.*, i, 1044.
KIDDER, G. W. AND DEWEY, V. C.—(1949) *J. biol. Chem.*, **179**, 181.
KIHLMAN, B.—(1952) *Symp. bot. upsaliens.*, **11**, 41. [Quoted by Boyland, E.—(1954) *Pharmacol. Rev.*, **6**, 345.]
KLEIN, M.—(1952) *J. Nat. Cancer Inst.*, **13**, 333.
LARSEN, C. D.—(1948) *Ibid.*, **9**, 35.
LAW, L. W.—(1950) *Cancer Res.*, **10**, 186.
LEITCH, A. AND KENNAWAY, E. L.—(1922) *Brit. med. J.*, ii, 1107.
LISSAUER.—(1865) *Berl. klin. Wschr.*, **2**, 403.
LOVELESS, A.—(1953) *Heredity*, supplement to vol. **6**, 293.
NEUBAUER, O.—(1947) *Brit. J. Cancer*, **1**, 192.
NOVICK, A. AND SZILARD, L.—(1951) *Cold Spr. Harb. Symp. quant. Biol.*, **16**, 337.
PITON, R.—(1929) *Arch. int. Méd exp.*, **5**, 355.
PULLINGER, B. D.—(1945) *J. Path. Bact.* **57**, 477.
QUASTEL, J. H. AND WHEATLEY, A. H. M.—(1933) *Proc. Roy. Soc., B*, **112**, 60.—(1934) *Biochem. J.* **28**, 1521.
RAPOPORT, I. A.—(1948) *Doklady Akad. Nauk. S.S.S.R.*, **61**, 713. [Quoted by Boyland, E.—(1954) *Pharmacol. Rev.*, **6**, 345.]
ROE, F. J. C.—(1956*a*) *Brit. J. Cancer*, **10**,61—(1956*b*) *Ibid.*, **10**, 72
*Idem* AND GLENDENNING, O. M.—(1956) *Ibid.*, **10**, 357.
*Idem* AND SALAMAN, M. H.—(1954) *Ibid.*, **8**, 666.—(1955) *Ibid.*, **9**, 177.
ROGERS, S.—(1954) *Proc. Amer. Ass., Cancer Res.*, **1**, 41.
ROSS, W. C. J., DAVIS, W., ROBERTS, J. J. AND EVERETT, J. L.—(1952) *Ann. Rep. Brit. Emp. Cancer Campgn.*, **30**, 25.
SALAMAN, M. H., AND GWYNN, R. H.—(1951) *Brit. J. Cancer*, **5**, 252.
*Idem* AND ROE, F. J. C.—(1953) *Ibid.*, **7**, 472.
SCHOENE, D. L. AND HOFFMANN, O. L.—(1949) *Science*, **109**, 588.

SKIPPER, H. E., BENNETT, L. L., EDWARDS, P. C., BRYAN, C. E., HUTCHISON, O. S., CHAPMAN, J. B. AND BELL, M.—(1950) *Cancer Res.*, **10**, 166.
*Idem*, MITCHELL, J. H., BENNETT, L. L., NEWTON, M. A., SIMPSON, L. AND EIDSON, M.—(1951) *Ibid.*, **11**, 145.
STEINER, P. E.—(1955) *Ibid.*, **15**, 632.
*Idem* AND FALK, H. L.—(1951) *Ibid.*, **11**, 56.
*Idem*, STEELE, R. AND KOCH, F. C.—(1943) *Ibid*, **3**, 100.
VALENTINER, W.—(1865) *Berl. klin. Wschr.*, **2**, 320.
WALPOLE, A. L., ROBERTS, D. C., ROSE, F. L., HENDRY, J. A. AND HOMER, R. F.—(1954) *Brit. J. Pharmacol.*, **9**, 306.
WARREN, C. O.—(1943) *Amer. J. Physiol.*, **139**, 719.
WHITE, D. G.—(1950) *Science*, **111**, 303.
WITKIN, E. M.—(1947) *Cold Spr. Harb. Symp. quant. Biol.*, **12**, 256.
WITTWER, S. H. AND SHARMA, R. C.—(1950) *Science*, **112**, 597.